# Conservative Management of Post-Operative Cerebrospinal Fluid Leak following Skull Base Surgery: A Pilot Study

**DOI:** 10.3390/brainsci12020152

**Published:** 2022-01-24

**Authors:** Aria M. Jamshidi, Ashish Shah, Daniel G. Eichberg, Ricardo J. Komotar, Michael Ivan

**Affiliations:** Department of Neurological Surgery, University of Miami Miller School of Medicine, Miami, FL 33136, USA; Ashah@med.miami.edu (A.S.); deichberg@jhsmiami.org (D.G.E.); Rkomotar@med.miami.edu (R.J.K.); Mivan@med.miami.edu (M.I.)

**Keywords:** acetazolamide, cerebrospinal fluid leak, lumbar puncture, endoscopic endonasal surgery

## Abstract

Background/aims: Iatrogenic CSF leaks after endoscopic endonasal transsphenoidal surgery remain a challenging entity to manage, typically treated with CSF diversion via lumbar drainage. Objective: To assess the safety and efficacy of high-volume lumbar puncture (LP) and acetazolamide therapy to manage iatrogenic CSF leaks. Methods: We performed a prospective pilot study of four patients who developed iatrogenic postoperative CSF leaks after transsphenoidal surgery and analyzed their response to treatment with concomitant high-volume lumbar puncture followed by acetazolamide therapy for 10 days. Data collected included demographics, intra-operative findings, including methodology of skull base repair and type of CSF leak, time to presentation with CSF leak, complications associated with high-volume LP and acetazolamide treatment, and length of follow-up. Results: Mean patient age was 44.28 years, with an average BMI of 27.4. Mean time from surgery to onset of CSF leak was 7.71 days. All four patients had resolution of their CSF leak at two- and four-week follow-up. Mean overall follow-up time was 179 days, with a 100% CSF leak cure rate at the last clinic visit. No patient suffered perioperative complications or complications secondary to treatment. Conclusion: Although our pilot case series is small, we demonstrate that a high-volume LP, followed by acetazolamide therapy for 10 days, can be considered in the management of post-operative CSF leaks.

## 1. Introduction

Iatrogenic cranial cerebrospinal fluid (CSF) leaks after trans-sphenoidal surgery remain a challenging, albeit common, complication to manage for both neurosurgeons and otolaryngologists. Unlike traumatic CSF leaks, which typically resolve with conservative measures (e.g., bedrest), iatrogenic CSF leaks tend to be refractory to these techniques. Consequently, invasive procedures, such as CSF diversion and revision surgery, are often warranted [1].

The role of perioperative lumbar drainage (LD) in preventing this complication continues to be debated [2,3,4]. LD has been used to reduce the risk of intraoperative cerebrospinal fluid leak during transsphenoidal surgery and has also been used to inject air or saline to promote the delivery of the suprasellar portion of some tumors [5]. However, LD placement still remains an invasive procedure with a 3%–5% complication rate, including infection, meningitis, radiculopathy and lumbago [4]. Furthermore, patients with LD are at an increased risk of deep venous thrombosis and other complications secondary to their relative immobility [6]. Other, less invasive modalities for the management of CSF leaks are needed.

Historically, acetazolamide has been used to treat and manage traumatic CSF leaks. A prospective trial of 36 patients with 42 CSF leaks was conducted to evaluate the difference in opening pressure on lumbar puncture 4 h after one 500 mg dose of acetazolamide [7]. Acetazolamide successfully reduced opening pressure from a mean of 32 to 21.9 cm H_2_O. The medication was considered well tolerated, though a formal analysis of adverse effects was not performed. Thus, acetazolamide represents a possible modality to manage mild iatrogenic CSF leaks after transsphenoidal surgery as well.

We sought to somehow combine these two treatment paradigms to manage our low-grade post-operative CSF leaks after endoscopic transsphenoidal surgery. We initially performed a lumbar puncture to measure the opening pressure in these patients, to rule out hydrocephalus and meningitis, and then started each patient on 10 days of acetazolamide to further reduce CSF production and pressure. This study is focused on the safety and proof of concept of this strategy. 

## 2. Materials and Methods

### Patient Selection

We conducted a single-institution retrospective analysis of prospectively collected data to evaluate the safety and efficacy of high-volume lumbar puncture and acetazolamide therapy for the management of iatrogenic cerebrospinal fluid leak following transsphenoidal surgery for tumor resection. Patient were accrued at our institution from June 2018 to June 2019 and their data maintained in an institutional database. 

The study was performed with the approval of the Institutional Review Board, and the consent process was waived, as it is a retrospective review of patient data stripped of identifying information. 

After undergoing a full endocrinologic workup and ophthalmologic evaluation, patients underwent a standard endoscopic endonasal transsphenoidal surgical approach for pituitary macroadenoma resection. Each patient had a CSF leak, intra-operatively. According to the grade of leakage as observed during endoscopic evaluation, we divided the patients into two groups: those harboring a low-grade leak, indicated by a small weeping leak confirmed by Valsalva maneuver without an obvious or with only a small diaphragmatic defect; and those with a moderate leak, indicated by a definite diaphragmatic defect and egress of CSF [8]. Each leak was repaired with a combination of Cygnus Solo^®^ (Vivex Biomedical, Miami, FL) dehydrated amniotic membrane (DAM) [9,10,11], acellular dermis, and free mucosal flap. None of the patients developed any signs or symptoms of meningitis; however, given the potential risk of this event, all patients received broad spectrum prophylactic antibiotics with vancomycin and a third-generation cephalosporin throughout the entirety of their inpatient admission. Post-operatively, each patient was observed for 48 to 72 h to monitor for diabetes insipidus and development of CSF leak prior to discharge, and was given strict instructions to avoid straining, bending, or Valsalva in the post-operative period. 

**Figure 1:**
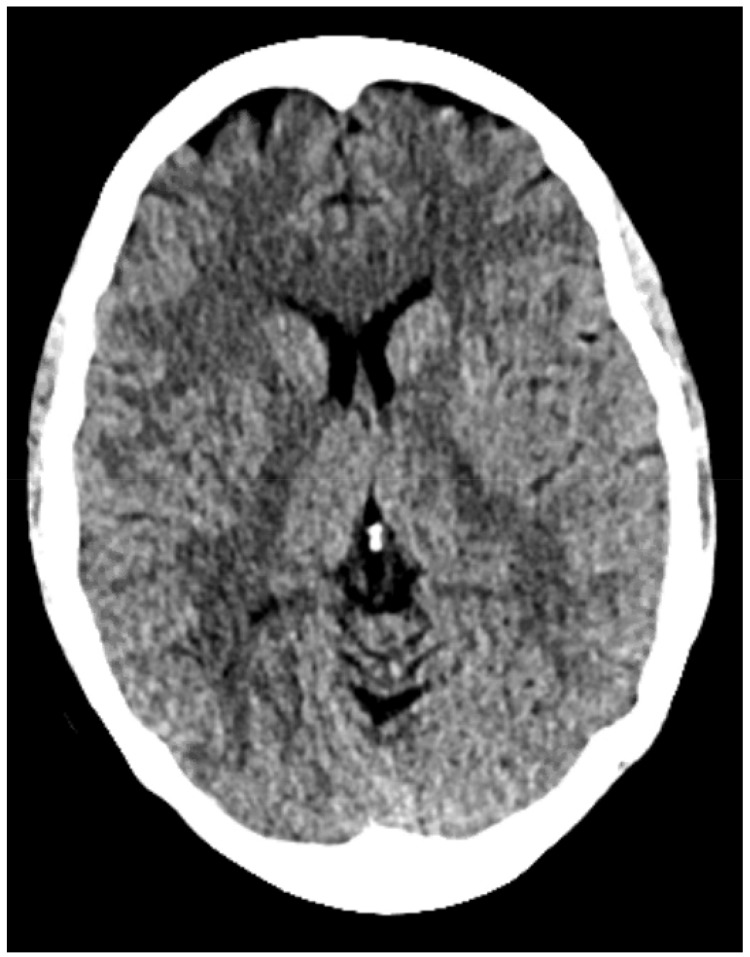
CT brain non-contrast performed after presentation for suspected CSF leak confirming no pneumocephalus.

Each patient subsequently presented at various time points after surgery with a low-grade CSF leak from the nares on a clinical exam that was found when the patient bent forward, confirmed by both a board-certified neurosurgeon and otolaryngologist. Cerebrospinal fluid was sent for B2-transferrin, which was confirmatory in all cases. Each patient then underwent a CT scan of the brain to rule out pneumocephalus (Figure 1) and hydrocephalus, and then subsequently underwent a high-volume lumbar puncture, where 30–40 cc of CSF was removed from the intrathecal space and sent for analysis, including Gram stain and culture. The patient was then started on acetazolamide 250 mg three times a day for 10 days and observed for two nights in the hospital [12].

Prior to discharge, each patient was evaluated to confirm cessation of their leak and subsequently discharged with strict instructions again to avoid Valsalva maneuvers and strenuous activity, given two weeks of stool softeners, and asked to maintain elevation of the head of their bed for two weeks. These patients were followed up in clinic by otolaryngology and neurosurgery to assess for the presence of CSF leak at two and four weeks, post-operatively. Clinical information was collected retrospectively.

Inclusion criteria comprised the following: (1) patients older than 18 years old; (2) patients with less than 1 cm^3^ of pneumocephalus on CT brain non-contrast (Figure 1); and (3) patients with only mild CSF leaks from the nose that could only be elicited when the patient bent forward. Patients were excluded from our analysis if they presented with signs/symptoms of meningitis as evidenced by clinical (high fever, meningismus, and nausea/vomiting) and laboratory analyses (blood cultures, and CSF profile/staining), or if they had high flow CSF leaks that would not be amenable to this conservative approach. Of note, the senior surgeon maintains a prospective log of all transsphenoidal surgeries done at our center; we included all patients who met the above criteria, regardless of whether they failed this conservative management or not. 

This study was performed in accordance with PROCESS guidelines [13].

## 3. Results

### Case Series

**Figure 2:**
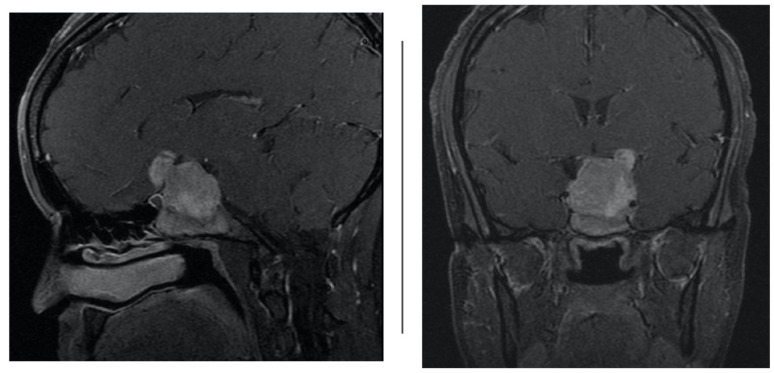
Sagittal and coronal T1 contrast-enhanced weighted magnetic resonance imaging (MRI) demonstrating large sellar and suprasellar pituitary macroadenoma extending into the cavernous sinus.

At our institution, four patients (*n* = 4) were assigned to acetazolamide therapy after they developed iatrogenic cerebrospinal fluid leak from transsphenoidal surgery for tumor resection from June 2018 to June 2019 by the senior neurosurgeon. Table 1 shows a summary of patient demographics. Average age was 42.5 years and average BMI was 31.6 (range 26.5–36.4). All four tumors were greater than 10 millimeters in size and had suprasellar extension (Hardy 3); in three of the four cases, the tumor compressed the optic chiasm and infiltrated the sphenoid sinus (Hardy 4). Further, in all four cases, the macroadenoma extended to or beyond the lateral aspects of the internal carotid artery, and in some cases extended into the superior and inferior cavernous sinus (Figure 2) compartment (Grade 3B). Average tumor volume was 21.24 [3]. Using the CSF leak grading system described by Esposito et al., all four patients had grade one to two CSF leaks, intra-operatively [8]. These leaks were repaired with a combination of dehydrated amniotic membrane, acellular dermis, and free mucosal flap based on the intraoperative judgement of the senior otolaryngologist. 

Mean time from surgery to onset of clinically detectable postoperative CSF leak was 6.75 days. All opening pressures on lumbar puncture were less than 20 mmHg, and CSF profiles were negative for meningitis. No complications directly attributable to acetazolamide or the high-volume lumbar puncture were encountered during treatment. At two- and four-week follow-up, all four patients denied any further leakage of CSF from the nares, confirmed by evaluation by a neurosurgeon and otolaryngologist. Mean overall follow-up time was 154.75 days.

## 4. Discussion

Acetazolamide is an established carbonic anhydrase inhibitor, well known for its use in the treatment of altitude sickness, glaucoma, and epilepsy. However, in addition to these indications approved by the Food and Drug Administration, acetazolamide also has several off-label uses [14]. In the neurosurgical practice, it is well known for its efficacy in management of idiopathic intracranial hypertension and CSF leak [15]. Acetazolamide decreases CSF flow by inhibiting 99.5% of carbonic anhydrase production in the choroid plexus, decreasing CSF production by as much as 48%, resulting in a reduction in intracranial pressure [16].

An open-label, multicenter, randomized controlled trail was conducted evaluating early (*n* = 28) versus late (*n* = 29) treatment with acetazolamide in adult and pediatric patients with, or at risk of, a CSF leak due to a skull base fracture. In the early treatment group, patients received acetazolamide with 48 h of admission, and had greater mean of CSF leak resolution rate within 14 days (100% versus 78% in the late treatment group), and a shorter mean CSF leak duration (2.2 versus 3.8 days in the late-treatment group. However, there is a dearth of literature with regards to the treatment of post-operative CSF leak with acetazolamide. 

Chaaban et al., published their results from 46 patients with 56 spontaneous CSF leaks managed over 5 years. Fifty-two CSF leaks (93%) were successfully repaired at first attempt. Of note, opening pressures via lumbar puncture increased significantly following closure of the skull base defect by 8 cm H_2_O. As such, 24 subjects (52%) were subsequently managed with acetazolamide therapy post-operatively. However, 2 of the 24 subjects managed with Diamox became intolerant of the medication, and ultimately required permanent CSF diversion with VP shunt placement. Notably, 22 of the 24 patients (92%) were successfully treated with acetazolamide for their elevated ICP [17].

In our series, we studied the use of acetazolamide with a concomitant high-volume lumbar puncture as salvage therapy to treat iatrogenic CSF leaks after transsphenoidal surgery for tumor resection. Patients were hospitalized for an average of two nights after undergoing lumbar puncture and were subsequently discharged. This stands in strong contrast to the typical prolonged hospital stays associated with lumbar drainage. Further, these patients did not suffer the common side effects of lumbar drainage, such as subdural hematomas or hygromas, meningitis, and low-pressure headache, which can be debilitating in some circumstances [18,19]. At both follow-up visits, two and four weeks after surgery, none of our patients endorsed continued CSF leak after initiation of our conservative management. On the last follow-up, we report a 100% CSF leak cure rate. No patients required either additional surgery for dural repair, or a lumbar drain for added CSF diversion. The added morbidity and mortality associated with these procedures, including bleeding, infection, and meningitis, amongst others, were thus avoided in this patient cohort. 

No complications directly attributable to acetazolamide or the high-volume lumbar puncture were encountered. We believe this treatment regimen transiently reduces intracranial pressure, and allows for normal dural scarring to occur, ultimately preventing further CSF leakage. 

## 5. Limitations

The present study has several limitations. First, our pilot study is inherently limited by a small sample size and the natural biases of a single-surgeon series. Second, there is no control group to compare traditional management with early skull base re-construction to our pilot group managed with acetazolamide and lumbar puncture. Finally, the authors acknowledge that there is no definitive way of determining if lumbar puncture had any bearing on the success of this treatment in this small cohort of patients, or rather, if the acetazolamide was the sine qua non reason for CSF leak resolution. However, these represent a novel approach to managing iatrogenic cerebrospinal fluid leak. To further study and confirm these results, we will use these initial positive data to justify a randomized control trial, looking at the efficacy of this management paradigm.

## 6. Conclusions

We report one surgeon’s case series of four patients who suffered from iatrogenic postoperative CSF leak following endoscopic endonasal surgery and were all successfully managed with high-volume lumbar puncture and acetazolamide therapy. This approach eliminates the morbidity associated with lumbar drainage, the classical neurosurgical practice to manage post-operative CSF leaks. With close follow-up, these patients can be managed in a relatively conservative fashion, eliminating the need for prolonged hospital stays, lumbar drainage, and additional surgery.

## Figures and Tables

**Table 1:** Demographics, pathology, time to CSF leak and management of four patients who experienced iatrogenic CSF leaks after trans-sphenoidal surgery for tumor resection.

Case #	Age	Gender	Pathology	Dimensions (ap × t × cc)	Pattern of Growth	Intraop. Leak Grade	Time to CSF Leak (Days)	Total Length of Follow-Up	Complications
1	26	F	Pituitary Macroadenoma	3.2 × 2.9 × 3.2 cm	Suprasellar Extension Hardy 4, Knosp 3B	Low	4	198 days	None
2	37	M	Pituitary Macroadenoma	2.1 × 2.1 × 2.2 cm	Suprasellar Extension Hardy 4, Knosp 3A	Low	10	330 days	None
3	42	F	Pituitary Macroadenoma	4.5 × 3.3 × 2.9 cm	Suprasellar Extension Hardy 4, Knosp 4	Moderate	8	53 days	None
4	65	F	Pituitary Macroadenoma	1.3 × 1.3 × 1.5 cm	Suprasellar Extension Hardy 3, Knosp 2	Moderate	5	38 days	None
